# Booster immunizations with DNA plasmids encoding HER-2/neu prevent spontaneous mammary cancer in HER-2/neu transgenic mice over life span

**DOI:** 10.1038/s41598-017-03286-8

**Published:** 2017-06-08

**Authors:** Mauro Provinciali, Alessandra Barucca, Fiorenza Orlando, Elisa Pierpaoli

**Affiliations:** Advanced Technology Center for Aging Research, Scientific Technological Area, IRCCS-INRCA, Ancona, Italy

## Abstract

Cancer vaccines are less effective at old than at young age because of immunosenescence. Besides, in preliminary observations we showed that the immunization with HER-2/neu DNA plasmid in transgenic young mice (standard immunization, SI) delays but not abrogate spontaneous mammary tumours progressively appearing during aging. In this study we evaluated whether booster immunizations (BI) of HER-2/neu transgenic mice with HER-2/neu DNA plasmids every 6 (ECD6), 3 (ECD3), or 1.5 (ECD1.5) months after SI induce a protective immunity that could be maintained over life span. The long term BI significantly improved the effect of SI increasing the number of tumour free mice at 110 weeks of age from 13% (SI) to 58% (BI). Both the number and the volume of tumour masses were reduced in BI than in SI groups. The protective effect of BI was associated with increased antibody production with isotype switching to IgG2a, augmented CD4 T cells, and increased *in vivo* cytotoxicity of HER-2/neu specific cytotoxic T lymphocytes, mainly in ECD1.5 and ECD3 groups. The transfer of sera from ECD1.5 mice to untreated HER-2/neu mice highly protected against tumour development than sera from SI mice. We conclude that BI induce a protective immunity effective over life span.

## Introduction

Studies conducted in various animal models have demonstrated that anticancer vaccination represents an efficacious approach to elicit a potent immune response and to induce immune memory against tumour antigens^1–3^. DNA immunization against mammary tumour represents an optimal experimental model to highlight the effectiveness of antitumour vaccines. Several studies of DNA vaccination have been performed in tumours over expressing HER2/*neu*, an oncogene coding for a transmembrane protein (p185neu) and belonging to the family of tyrosine kinase growth factor receptors. HER2/*neu* gene amplification and consequent over expression of HER2/*neu* receptor have been observed in a significant proportion of human tumours, particularly in breast cancer, and are intimately associated with malignant phenotype and aggressiveness of the malignancy^4–7^.

The experiment models performed until now have clearly demonstrated that the efficacy of antitumour vaccination is dependent on the immune competence of the host^8, 9^. We and others have reported that cancer vaccines are less effective at old than at young age because of immunosenescence^10–15^. In fact, one of the aspects of immunosenescence is an impaired number and repertoire of naïve T and B cells^16^, a situation that implies inadequate capacity of mounting an optimal protective response. In our experience, the immunization with HER2/*neu* DNA plasmids completely protected young but not old mice against a lethal challenge of mammary HER2/*neu* carcinoma cells^11, 13^.

A good model for studying cancer immunoprevention through DNA vaccination is that of HER-2/*neu* transgenic mice, an *in vivo* experimental model of mammary cancer that strongly emulates human disease^17^. In these mice, HER2/*neu* DNA immunization in young age, completely prevents the spontaneous development of mammary tumours, which normally occurs in 100% of animals at 5–6 months of age^13^. However, in preliminary experiments, we observed that the standard immunization procedure with DNA coding for HER-2/neu in young transgenic mice delays but not abrogate the appearance of mammary tumours, since tumour masses progressively appeared after 40 weeks of age and more than 80% of mice were bearing tumour masses after 90 weeks of age. These effects could be explained by changes arising with the immunosenescence that render the “old” immune system unable to respond efficiently to the antigenic stimuli, and/or to the short-term protection of immunization strategies, that, once activated in young age, lose their effectiveness during aging.

Among the possible strategies to improve vaccine efficacy in old age is the use of booster vaccinations. This approach, mainly studied until now in the field of infectious diseases, is based on the fact that, although elderly people are able to mount a T cell response after vaccination, they also exhibit an impaired long-term immune response^18^. In fact, it has been shown that the time elapsed since last vaccination has a significant effect on the antibody titres and can be essential for a protective response^19^.

To evaluate the possibility to prevent the age-dependent appearance of mammary tumours in HER-2/*neu* transgenic mice, we elaborated a protocol of immunization performed in young age that could be effective until very old ages, overcoming the defects linked to the immunosenescence.

Using the HER-2/*neu* transgenic mouse model of mammary cancer we analyzed in this study whether the immunization with DNA plasmids encoding HER-2/*neu* in young age induces a protective immunity that could be maintained over life span through BI.

## Results

### Kinetics of tumour growth

HER-2/*neu* transgenic mice, which spontaneously develop mammary carcinomas, were treated according to an established standard protocol of immunization and then divided in four groups, three of which received, starting at a young age, BI until they achieved old age. These groups were vaccinated repeatedly and regularly every 6 (ECD6), 3 (ECD3), or 1.5 (ECD1.5) months.

**Figure 1:**
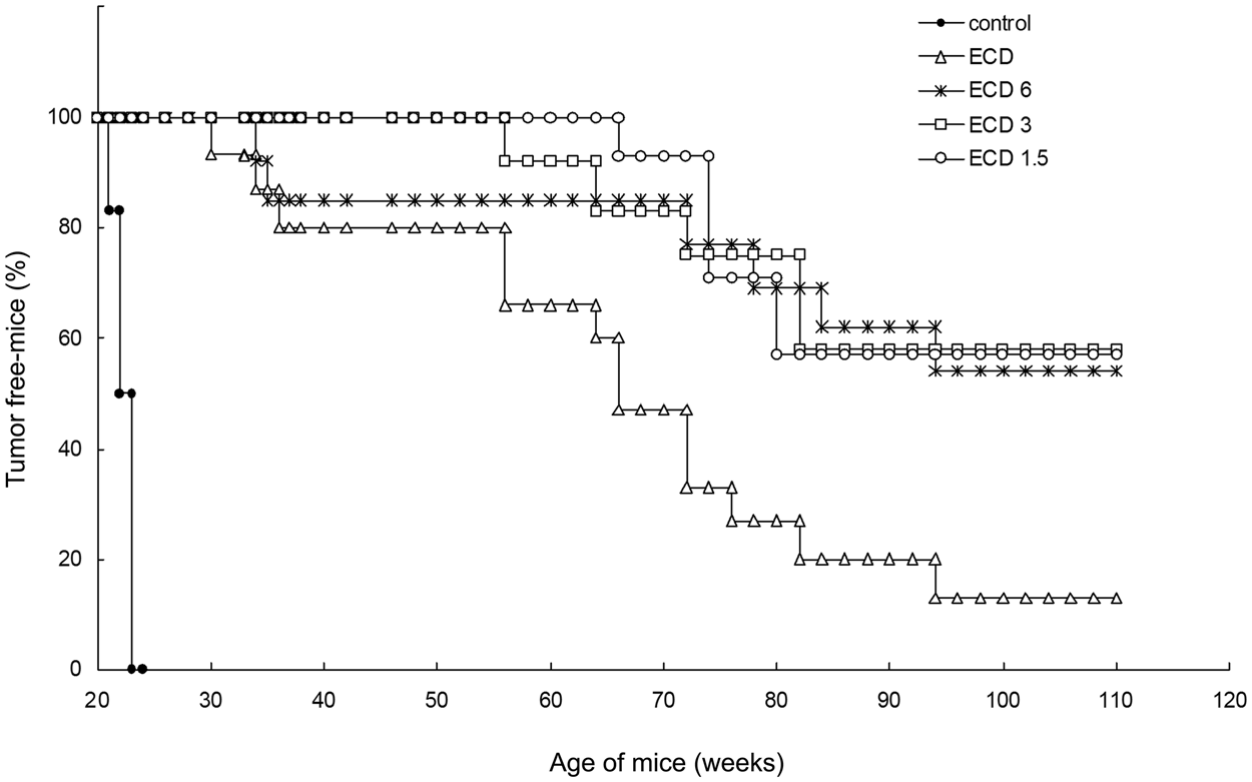
Effect of BI on tumour incidence. After SI, three groups of mice (15 mice/group) received BI every 6 (ECD6), 3 (ECD3), or 1.5 (ECD1.5) months. A control group received pCMV. Data shown are representative of one of two independent experiments. Difference in tumour incidence, as assessed by the Mantel–Haenszel log-rank test, was significant between ECD versus Control (p < 0.001) and ECD1.5, ECD3, and ECD6 versus control or ECD (p < 0.001).

As shown in Fig. 1 the immunization through different vaccination schedules differently affected the growth of spontaneous tumours in transgenic mice. In controls, the first tumour mass appeared at around week 20 of age and, by week 25, 100% of mice were tumour bearers. In animals immunized with pCMV-ECD by SI protocol, the time of tumour onset was delayed, with 10% of mice with the first tumour at week 30, 20% of mice developing the first tumour at around weeks 35, and a following progressive increase of tumour incidence with 87% of mice with tumour at week 110 (*p* < 0.001 versus control group). Long-term BI significantly improved the outcome of standard vaccination without significant differences among the three different immunization schedules (p < 0.01, p < 0.004, and p < 0.001, for ECD6, ECD3, or ECD1.5, respectively vs. ECD). When the three groups receiving BI were compared, the protection of mice against tumour appearance was more effective until week 75 for the group ECD1.5, while after week 75 all groups had a similar trend and at week 110 of age about the 58% of mice were still tumour free (*p* < 0.001 versus control group and SI).

**Figure 2:**
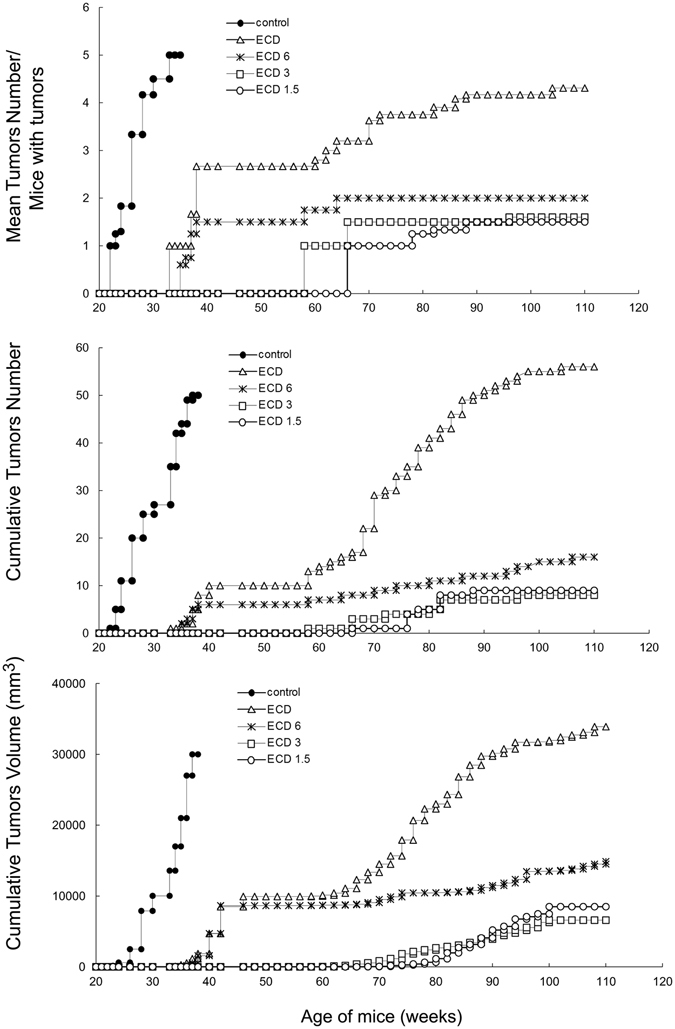
Effect of BI on the mean tumours number/mice with tumours (top), the cumulative tumours number (middle), and the cumulative tumours volume (bottom). All groups included 15 animals. Data shown are representative of one of two independent experiments. Statistically significance is reported in the results.

The protective effect of the vaccine was also assessed by monitoring the mean tumours number per mice with tumours, the cumulative tumours number per group, and the cumulative tumours volume per group. As shown in Fig. 2 (top), the reduction of tumour incidence obtained in mice treated with SI or BI was associated with a decrease of the number of tumour masses in comparison with control mice. In all BI-treated mice groups, a significantly lower tumour multiplicity was found in comparison with SI (*p* at least < 0.05). Mice of ECD1.5 or ECD3 groups showed a lower number of tumour masses when compared to mice of ECD6 group which was significant from week 38 to 64 for ECD1.5 (p = 0.05) or from week 38 to 56 for ECD3 (p = 0.05). The kinetics of the cumulative number of tumour masses per group (Fig. 2, middle), was similar to that of the mean number of tumour masses. The cumulative tumours volume per group (Fig. 2, bottom) was lower in BI-treated mice than in SI (ECD group) from week 65 for ECD6 (p < 0.05) and from week 45 for ECD3 or ECD1.5 groups (p < 0.05).

### Humoral Immunity

**Figure 3:**
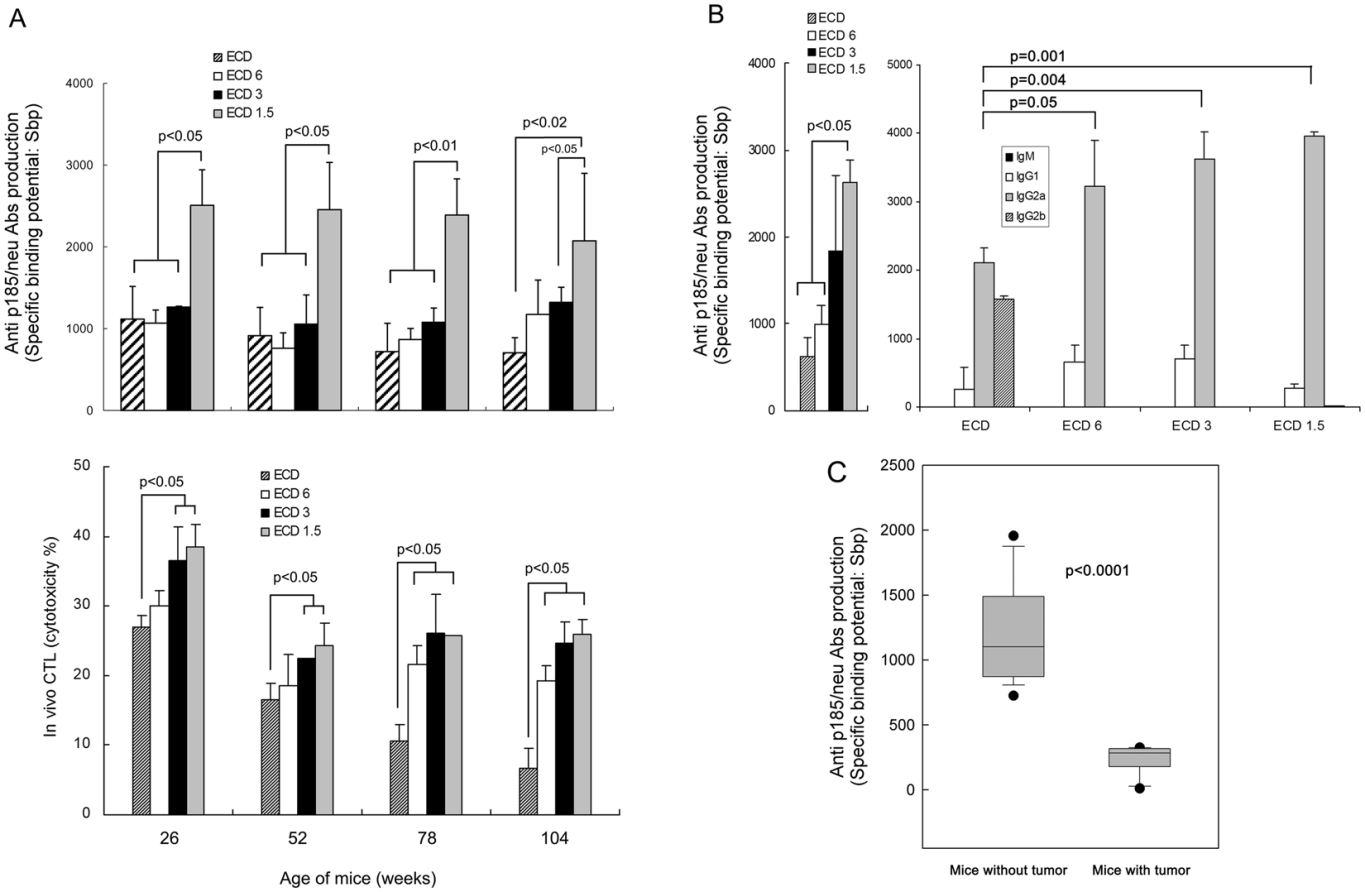
Presence of anti p185neu Abs and *in vivo* cytotoxicity in HER-2/*neu* transgenic mice after BI. Sbp of sera to p185 protein (**A**, top) or *in vivo* cytotoxicity (**A**, bottom) from three animals of SI and BI groups were analysed at all time points reported. The ability of sera to bind p185^neu^ (Sbp) was evaluated by flow cytometry as reported in M&M. For cytotoxicity, immunized mice of different age were injected with CFSE stained splenocytes from naıve transgenic mice pulsed with either HER-2 immunodominant or irrelevant peptides and were analyzed by flow cytometry as detailed in M&M.; anti-p185*neu* IgM or IgGs isotytpes were analysed in three mice of SI and BI groups at 39 weeks of age (**B**); p185 protein in 104 weeks old-mice of ECD1.5 group with or without tumour are shown (**C**).

As shown in Fig. 3A (top), the production of anti-p185neu antibodies in ECD6 and ECD3 groups was generally increased but not significantly different from the production of antibodies in animals treated with SI. Anti-p185neu antibodies were significantly increased in ECD1.5 group in comparison with ECD, ECD6, or ECD3 groups (p at least < 0.05 where indicated) (Fig. 3A, top). In mice vaccinated with SI, anti-p185neu antibodies were mainly of the IgG2a and IgG2b subclasses, with a minor IgG1 component (Fig. 3B). Sera from the groups with BI showed a significantly higher titer of IgG2a (p = 0.001, p = 0.004, and p = 0.05 for ECD1.5, ECD3, and ECD6 vs. ECD) and reduced levels of IgG2b (p < 0.001 vs. ECD) than those observed in mice vaccinated with SI. As shown in Fig. 3C, the production of antibodies in mice from ECD1.5 group at 104 weeks of life was significantly higher in mice without tumour in comparison with mice with tumour (p < 0.001).

### Cell-mediated Immunity

Figure 3A (bottom) reports the mean of *in vivo* cytotoxicity observed in the spleen of immunized mice. As shown, the CTL activity of mice from ECD3 or ECD1.5 groups was significantly higher than that of SI at all time points examined (26, 52, 78, and 104 weeks of age, p at least < 0.05). The CTL activity of mice from ECD6 group was significantly increased over SI only at 78 and 104 weeks of age (p at least < 0.05; Fig. 3A, bottom).

### Immune phenotype

**Figure 4:**
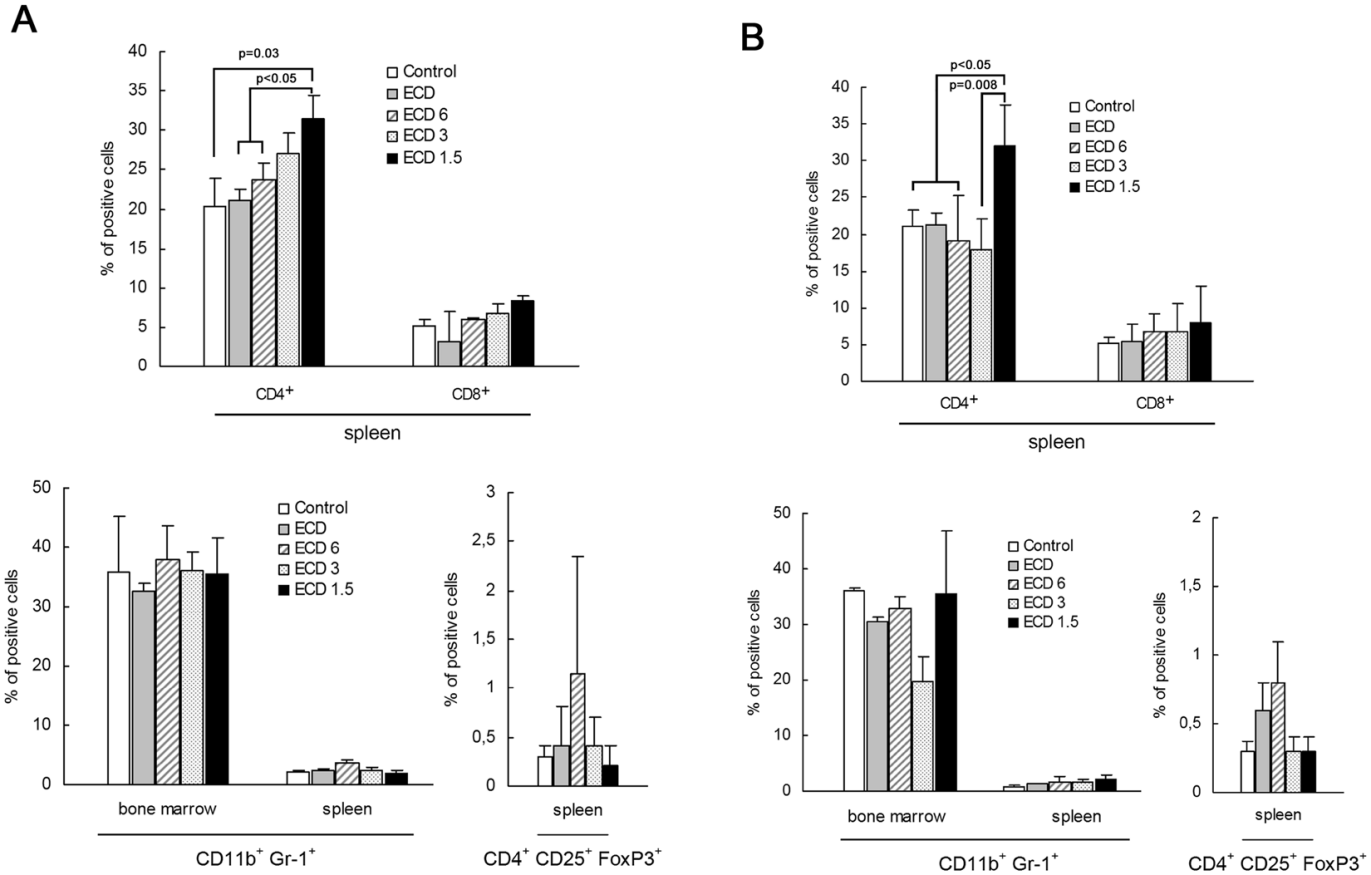
Effect of BI on the leukocyte phenotype. Spleen or bone marrow cells from 76 (**A**) or 96 (**B**) week-old immunized mice were stained with moAbs specific for the following antigens: CD8a (Ly-2), CD4 (L3T4), CD4^+^CD25^+^Foxp3^+^ (regulatory T cells), and CD11b^+^Gr-1^+^ (myeloid-derived suppressor cells) and analyzed by flow cytometry. Three animals per group were analysed. When present, difference in the percentage of leukocyte among groups was reported in the figure.

As shown in Fig. 4A, the percentage of CD4 T lymphocytes in 76 week-old mice was significantly increased in ECD1.5 group in comparison with control mice (p = 0.03) or ECD and ECD6 groups of mice (p < 0.05). A similar increase of CD4 T cells was obtained in 96 week-old animals in ECD1.5 in comparison with controls, ECD, or ECD6 (p < 0.05), or with ECD3 (p = 0.008) (Fig. 4B). Although CD8 T cells showed a trend towards an increased representation in groups treated with BI, the values were not significantly different in comparison with SI. No significant difference was observed at the level of both CD11b^+^Gr-1^+^ myeloid-derived suppressor cell and CD4^+^CD25^+^FoxP3^+^ regulatory T cell populations in the different groups of mice examined.

### Effect of adoptive transfer of sera

**Figure 5:**
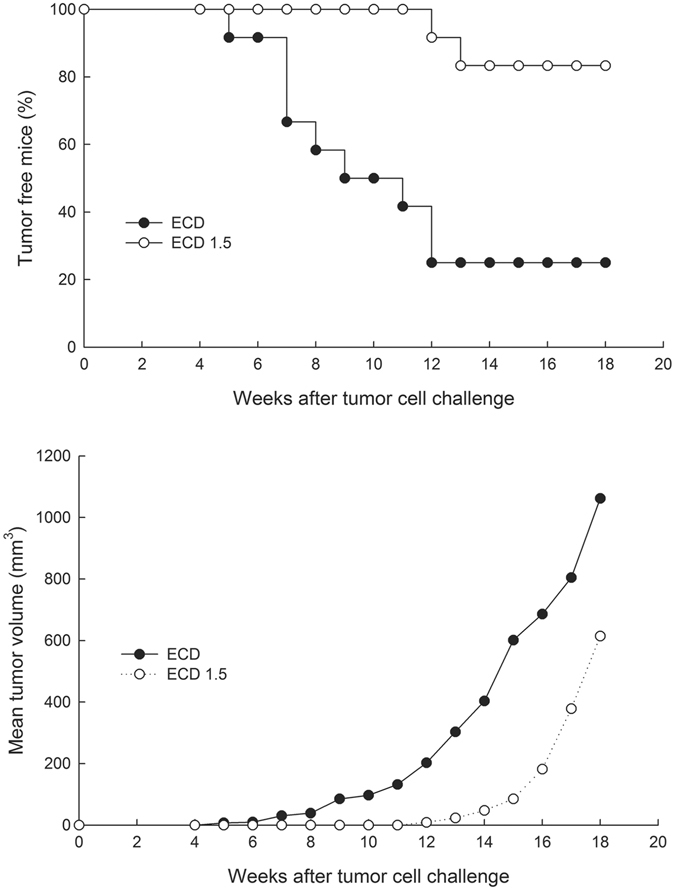
*In vivo* anti-tumour effect of anti-p185 sera from immunized mice. The capacity of anti-p185neu sera to interfere with the development of p185- overexpressing 202/1 A tumours cells is shown. Sera from 56 week-old ECD or ECD1.5 mice were i.p. injected in 8 weeks-old FVB/N mice (12 mice/group); 24 h after mice were s.c. challenged with N202/1 A tumour cells. Effect of sera from SI or ECD1.5 on tumour incidence (top) or mean tumour volume (bottom) are shown. Difference in tumour incidence, as assessed by the Mantel–Haenszel log-rank test, was significant between ECD vs. ECD1.5 (*p* < 0.001). Difference in mean tumour volume was assessed by T test.

Since the analysis of anti-p185neu antibodies indicated a substantial increase of antibodies with a switch from IgG1 and IgG2b to IgG2a phenotype in mice from ECD1.5 group, we investigated the protection afforded by the transfer of sera from mice of ECD1.5 group in comparison with the sera of mice treated with SI in untreated young FVB female recipients. As shown in Fig. 5 (top), transfer of sera from mice of ECD 1.5 group provided significant inhibition of the growth of N202/1 A tumour cells, with a higher percentage of tumour free mice than with sera from mice treated with standard vaccine (p = 0.001). Furthermore, in the low number of young mice which were unprotected by ECD1.5 sera, a decreased mean tumour volume was present in comparison with mice with tumours treated with sera from SI group (p at least < 0.05 from week 12 to week 18, Fig. 5, bottom). No protection followed the transfer of serum from unvaccinated mice.

## Discussion

Antitumour vaccines have been extensively studied in the last decades and, among these, DNA vaccines have offered new approaches for the prevention and therapy of cancer disease^20, 21^. Clear evidence has been shown that the efficacy of antitumour vaccination is dependent on the immunocompetence of the host and that no protection against tumour challenge was obtained in physically or chemically immunosuppressed host^8, 9^. In this context, we and others demonstrated that age-related alterations of the immune system are responsible for the lower effectiveness of cancer vaccines in old ages^12–14, 22^. The immunization with either tumour cells engineered for the production of cytokines^10^ or with plasmid DNA encoding HER2/neu oncoprotein^11^ induced a lower protective immunity in old mice as compared to young animals. The poor objective response was associated both to a reduced leukocyte infiltrate in the tumour area, with nearly absent CD4 or CD8 T cells^10^, and to an impaired immune activation after intramuscular DNA immunization encoding HER-2/neu in old animals^11^. The antibody production and the proliferative capacity of lymphocytes were both lower in old than in young mice and the activity of cytotoxic T lymphocytes was not found in old animals^11^. The lower effectiveness of cancer vaccines in old ages has been related to the lack of naïve T cells, the impairment of activation pathways of T cells and antigen-presenting cells, and to age-related changes in the tumour microenvironment^22, 23^. These age-associated alterations render very difficult the induction of primary specific immune responses.

HER-2/neu transgenic mice represent a model of spontaneous mammary cancer that strongly emulates human disease. This experimental model has been widely used in recent years to test the effectiveness of *in vivo* anticancer strategies based on immunization with plasmid DNA or supplementation with potential anticancer compounds^24–27^.

Our preliminary data obtained in these transgenic mice showed that the HER-2/neu immunization through a protocol which consisted of two administrations carried out in young age (at 6 and 8 weeks of age, standard immunization, SI) delays but not abrogate the spontaneous appearance of mammary tumours, since tumour masses progressively appeared after 40 weeks of age and more than 80% of mice were bearing tumour masses after 90 weeks of age. Because of these defects, one alternative strategy to protect against breast cancer until old ages, may be to develop a BI protocol that, once started in young age, may be repeated throughout life span. With these premises, we conducted a study aimed at both to confirm our preliminary observation of the long-term inefficacy of SI in young age and to evaluate whether a protocol of BI performed in young age and repeated during life-span could be effective to enable HER-2/neu transgenic mice to become old and tumour free. In this context, mice were immunized in young age when their immune system is still able to generate a tumour-specific immune memory and the immunization has been repeated every 1.5, 3, or 6 months during life-span of mice.

The results of the study clearly demonstrated that: a) HER-2/neu DNA SI did not protect transgenic mice over life span; b) the BI with DNA plamids encoding HER-2/neu was able to significantly improve the effect of SI; c) the protective effect of BI was associated with increased humoral and cell-mediated anti-HER-2/neu specific immunity.

The first result of the study confirmed our preliminary observation showing that SI in young age does not protect mice against the development of spontaneous mammary tumours until old ages. In effect, the appearance of tumours started after 30 weeks of age and progressively increased afterword, with 87% of mice bearing at least a mammary tumour at week 110. Most of the studies aimed at verifying the efficacy of cancer vaccines have been generally conducted in young/adult ages; in these studies, immunized individuals were monitored for the appearance of tumour growth for short periods of time, usually until tumours grow in 100% of controls. Then, these studies did not take into account the influence the age-related immune system alterations may have on the effectiveness of cancer vaccines. Our study clearly demonstrates that long-term follow-up is necessary to evaluate fully the potential or lack of potential of tumour vaccines. The second and most important result of the study was to demonstrate that the immunization of HER-2/neu transgenic mice with DNA plasmids encoding HER-2/neu oncoprotein in young age induced a protective immunity that can be maintained during life-span through BI and that can significantly prevent the development of mammary cancers spontaneously appearing in HER-2/neu transgenic mice. Whereas in 100% of unvaccinated mice at least one mammary tumour developed within the 25^th^ week of life, around 58% of mice receiving a BI protocol was tumour free at 110 weeks of life. This data is significantly higher than that obtained with SI that protects only 13% of transgenic mice from tumour development at 110 weeks of age. BI not only increased the number of tumour-free mice but also reduced the mean and the cumulative tumours number, and the cumulative volume of tumour masses.

Two papers, for our knowledge, have reported the effectiveness of repeated HER-2/neu DNA immunization. In the paper of Quaglino *et al*.^28^ all Balb/c HER-2/neu transgenic mice that received four DNA electroporation courses at weeks 10–12, 20–22, 30–32, and 40–42 were tumour free at 1 year of age. In a more recent paper, Curcio *et al*.^29^ reported that transgenic BALB-neuT664V-E mice, once vaccinated at a 10- week interval with a HER-2/neu DNA plasmid by electroporation, remained tumour free until they were 15-mo old. Our present study represents the first analysis of the effectiveness of booster DNA immunizations until very old ages of mice, following animals over their life-span until more than two years of age. The experimental design we used provides new knowledge on the long term effectiveness of BI against HER-2/*neu* oncogene. In ECD6 group, the first booster happened at approximately 35 weeks of age. Until this time point there was no difference in treatment between ECD and ECD6. In line with this observation, there were no changes at this time in ECD and ECD6 groups in the percentage of tumour-free mice (Fig. 1) as well as in the immune parameters analyzed (Fig. 3). In our experimental mouse model, through both the ECD1.5 and the ECD3 groups were 100% tumour free at 1 year of age (similarly to what reported in Quaglino 2004 or Curcio 2008), soon after, however, the tumours began to develop in some mouse despite the BI. Then, even if the long term BI greatly ameliorated the effectiveness of SI, it did not determine a complete protection during aging since at least part of mice developed tumour masses when really old. This would imply that the BI schedule improved but not completely corrected the immunological defects appearing with immunosenescence. However, the data reported in this study, clearly demonstrate that the memory immune responses have been efficiently activated by BI independently of age and were effective for a long time.

Although all the three BI protocols determined a better protection than SI against the age-related development of spontaneous mammary tumours in transgenic mice, the cumulative number of data seems to show a greater effectiveness of the ECD1.5 group, followed by the ECD3 group. In fact, the protection against tumour development was higher until about the 70^th^ week for the group ECD1.5 and only after this time the three groups conformed their curves of tumour appearance. Furthermore, a lower number and volume of tumour masses was particularly evident in ECD1.5 and ECD3 groups. Also the measurement of antibody-dependent and cell-mediated immunity suggested for the better efficacy of these two immunization schedules. The production of specific antibodies seems to play a pivotal role in the protection elicited by BI. An increased antibody production was particularly observed in ECD1.5 group in which the anti-p185 antibody titer was maintained constant during the life of animals. Furthermore, BI strongly induced an isotype switch to IgG2a, the most effective for antitumour activity against HER-2/*neu* tumours^30^. This switch may explain at least in part the better protection of antibody response even in ECD6 and ECD3 groups in which the antibody production was not significantly increased in comparison with SI. The relevant role of antibodies in tumour protection is also demonstrated by the higher antibody titer in mice without tumour than in those with tumours in ECD1.5 group (Fig. 3) and by the protection induced by sera from ECD1.5 immunized mice in unvaccinated mice challenged with HER-2/neu tumour cells (Fig. 5). Cell mediated immunity was also involved in the protective effect of BI as demonstrated by the increased *in vivo* cytotoxicity of HER-2/neu specific cytotoxic T lymphocytes (Fig. 3). The increase of cytotoxicity was significant when compared to ECD group only in ECD1.5 and ECD3 groups until the age of 52 weeks; afterwards, the increase of cytotoxicity became significant also in ECD6 group. This fact was mainly related to the progressive decline of cytotoxicity occurring in ECD group after week 26. The mechanisms involved in the decline of cytotoxicity present in ECD1.5 and ECD3 groups after week 26 remain to be investigated. A higher percentage of CD4 T cells also attested for the biologic advantage offered by ECD1.5 immunization. Our opinion is that the significant increase in CD4 T cells found in ECD1.5 group, both in 76 week-old and 96 week-old mice, may be associated with the repeated immunizations which could maintain a sufficient number of memory T cells able to activate cytotoxic immune responses. We further analysed the representation of both T reg cells and MDSCs in our study. Both these suppressor cell populations have been shown to restrict vaccine-induced T cell responses in different experimental models^31, 32^. A potential influence of suppressor cells on the effectiveness of immunization is particularly suspected in advanced ages, since it is generally accepted that both regulatory T cells and MDSC increase with aging^33, 34^. On the other hand, cancer immunization might also modulate the suppressor cells representation; in this context, it has been recently shown that an anti melanoma DNA vaccine induced a robust and broad immune response and inhibited MDSCs and tumour growth^35^. Interestingly, in our study, the inability of BI to modulate the number of either T reg cells or MDSCs, suggests that the beneficial effect of BI was not reduced by a potential BI-related induction of suppressor cells, and that the BI effectiveness was not related to a reduced representation of these two suppressor populations.

In conclusion, the data reported in this study demonstrated the possibility to significantly improve the long term protection offered by plasmid DNA immunization by BI. This strategy permits to activate a persistent antibody and cell-mediated immunity which prevents the spontaneous development of mammary tumours in HER-2/*neu* transgenic mice over their life span.

## Methods

### Mice

FVB/*neu*-NT transgenic female mice for the activated rat *neu* oncogene and FVB/N female mice were obtained from Charles River (Hollister, CA) and were maintained under specific pathogen–free conditions under a standard light/dark regimen (12 h light/12 h darkness) in our animal facilities. Mice were housed in plastic non–galvanized cages and fed with standard pellet food (Nossan, Italy) and tap water ad libitum. All methods were carried out in accordance with the guidelines and regulations of the Italian legislation defined in the D.L. No. 116 of 27 January 1992. All experimental protocols were approved by Italian Health Ministry (Project “Study of anticancer effect of DNA vaccines in spontaneous or challenged tumours mouse models” Prot. N°1IM/01-11, PI MP).

### Plasmid DNA and immunization protocol

pCMV-ECDTM plasmid, encoding extracellular and transmembrane region of HER2/*neu* antigen under the control of the CMV early promoter/enhancer has been kindly provided by Dr Augusto Amici from the Camerino University; pCMV empty vector was used to treat control mice. Large scale preparation of plasmid DNA was carried by Giga kit (Qiagen) according to the manufacturer’s instructions. Two months old FVBneu-T female were randomly selected and immunized against HER-2/*neu* by intramuscular delivery of pCMV-ECDTM plasmid followed by electroporation procedure; pCMV “empty” vector was used to treat mice of control groups.

The DNA was suspended at a concentration of 1μg/μl in sterile water containing 6 mg/ml poly-L-glutammate and 150 mM NaCl; 50 μg of DNA were given to each animal through an injection into each tibial muscle followed by an electric pulse (200 V/cm, 25 msec/3 times) using the ECM 830 field generator (BTX Division, Genetronix).

Experimental design included a group of animals that was treated according to an established standard protocol of immunization, which consisted of two administrations carried out at 6 and 8 weeks, and three groups of animals that received, starting at young age, booster immunizations until they achieved old age. The three groups were vaccinated repeatedly and regularly every 6 (ECD6), 3 (ECD3), or 1.5 (ECD1.5) months (﻿see Supplementary information). All immune analyses were performed about 30–35 days after last immunization. For each of the above groups a control group immunized with the “empty” vector (pCMV plasmid) was prepared. Each group consisted of 15 mice. The experiment was repeated two times to allow for the evaluation of tumour growth and the study of immunological parameters.

### Evaluation of tumour growth

Immunized mice were evaluated for the appearance of spontaneous mammary tumours. The incidence and growth of tumours were checked twice weekly and the neoplastic masses were measured with calipers in the two perpendicular diameters. Mice with no evidence of tumour at the end of the observation period were classified as tumour-free whereas mice with a tumour of at least 3 mm mean diameter were classified as tumour bearers. Tumour volume was assessed with the following formula: 1/2(length × width^2^). The mean number of tumour masses/mice with tumours, the cumulative number of tumour masses, and the cumulative volume of tumour masses, were also registered. All mice bearing neoplastic masses exceeding 10 mm mean diameter were killed for humane reasons.

### Preparation and culture conditions of spleen cells

Spleen was teased through a 60-mesh sieve in Ca2+-and Mg2+-free phosphate-buffered saline (PBS, GIBCO, Gaithersburg, MD, USA) solution. Spleen cells were then fractionated on lympholyte M (Cedarlane, Canada) and mononuclear cells separated by density gradient centrifugation (500 g, 20 min). Cells from the interface of the gradients were washed twice with PBS and resuspended in RPMI 1640-containing penicillin (100 U/ml) and streptomycin (100 μg/ml).

### Immune phenotype

Single cell suspensions were obtained from spleen or bone marrow by flushing as previously reported^36^ in 76 and 96 week-old mice. The cells were stained with moAbs specific for the following antigens: CD8a (Ly-2), CD4 (L3T4), CD4^+^CD25^+^Foxp3^+^ (regulatory T cells), and CD11b^+^Gr-1^+^ (myeloid-derived suppressor cells) (all from Miltenyi Biotec, Germany). After staining, the cells were analysed through a Coulter Epics XL flow cytometer.

### Cytofluorimetric evaluation of anti-rat p185^neu^ antibodies

Serum of immunized mice was collected according to the protocoland stored at −80 °C until use. The ability of sera to bind p185^neu^ was evaluated by flow cytometry. N202/1 A cells (a tumour cell line established *in vitro* from a carcinoma that spontaneously arose in FVB/N mice carrying the rat HER-2/neu proto-oncogene and that expresses high levels of p185^neu^) were stained in a standard indirect immunofluorescence procedure with control or immune sera. The analysis of antibodies isotype was performed at 39 weeks of age. FITC-conjugated rabbit anti-mouse Ig or anti-mouse IgGM, anti-mouse IgG1, anti-mouse IgG2a and anti-mouse IgG2b (all from eBioscience) were used as second step Ab. The cells were analysed using a Coulter Epics XL flow cytometer.

### In vivo cytotoxic assay

*In vivo* cytotoxic assay was performed in 6 month-old mice and, subsequently, every 6 months. To generate differentially labelled target cells, splenocytes from naïve mice were incubated with either high (10μM, CFSE^high^) or low (2 μM, CFSE^low^) concentrations of CFSE (Cell Trace CFSE Cell Proliferation Kit, Molecular Probes). The CFSE^high^ cells were pulsed with the neu peptide, PDSLRDLSVF, and the CFSE^low^ cells was pulsed with the irrelevant peptide, RPQASGVYM. Both CFSE^high^ and CFSE^low^ were co-injected I.V. at a ratio of 1:1 in mice. Sixteen hours after injection, mice were sacrificed and residual CFSE^high^ and CFSE^low^ target cells remaining in recipients were analyzed in spleen by flow cytometry. Percent of cytotoxicity was calculated as follows: % cytotoxicity = 100 − [(neu peptide pCMV ECDTM/irrelevant peptide pCMV ECDTM)/(neu peptide pCMV/irrelevant peptide pCMV)] × 100.

### Adoptive transfer of sera

In order to test the ability of anti-p185neu sera to interfere with the development of p185neu-overexpressing tumour cells, 8 week-old FVB/N female mice received sera from ECD1.5 group or from mice treated with standard immunization. Sera were harvested from 56 weeks-old ECD1.5 and ECD mice. In total, 150μl of pooled sera of mice belonging to each one of the treatment groups was injected intraperitoneally in each animal (five animals/treatment sera). At 24 h after the treatment with sera, mice were s.c. challenged with 10^5^ N202/1 A tumour cells and successively monitored to register the kinetic of development of injected tumours.

### Statistics

Differences in tumour incidence were evaluated by the Mantel–Haenszel log-rank test. Differences in tumour multiplicity and tumour volume were evaluated by Student’s *t* test. Differences in immune parameters were evaluated by parametric (Student’s t-test) or non-parametric (Mann–Whitney) tests according to the distribution of the data. Differences were considered statistically significant when p ≤ 0.05. The statistical analysis was performed with Systat 10 (SPSS Inc) and SigmaStat software version 1.03 (Jandel Scientific, Germany).

## Electronic supplementary material

Supplementary Information

## References

[1] Finn OJ, Forni G (2002). Prophylactic cancer vaccines. Curr. Opin. Immunol..

[2] Quaglino E (2002). Immunological prevention of spontaneous tumours: a new prospect?. Immunol. Lett..

[3] Cavallo F, Forni G (2009). Recent advances in cancer immunotherapy with an emphasis on vaccines. Exp. Rev. Vaccines.

[4] Slamon DJ (1987). Human breast cancer: correlation of relapse and survival with amplification of the HER-2/neu oncogene. Science.

[5] Hynes NE, Stern DF (1994). The biology of erbB2/neu/HER2 and its role in cancer. BBA.

[6] Ross JS (2009). The HER-2 receptor and breast cancer: ten years of targeted anti–HER-2 therapy and personalized medicine. Oncologist.

[7] Yarden, Y. Biology of HER2 and its importance in breast cancer. *Oncology* **495**, 61(Suppl 2), 13 (2001). 10.1159/000055396 11694782

[8] Colombo MP, Forni G (1994). Cytokine gene transfer in tumour inhibition and tumour therapy: where are we now?. Immunol. Today.

[9] Cavallo F (1997). Antitumour efficacy of adenocarcinoma cells engineered to produce interleukin 12 (IL-12) or other cytokines compared with exogenous IL-12. J. Natl. Cancer Inst..

[10] Provinciali M, Argentati K, Tibaldi A (2000). Efficacy of cancer gene therapy in ageing: adenocarcinoma cells engineered to release IL-2 are rejected but do not induce tumour specific immune memory in old mice. Gene Ther..

[11] Provinciali M, Smorlesi A, Donnini A, Bartozzi B, Amici A (2003). Low effectiveness of DNA vaccination against HER-2/ neu in ageing. Vaccine.

[12] Lustgarten J, Dominguez AL, Thoman M (2004). Aged mice develop protective antitumour immune responses with appropriate costimulation. J. Immunol..

[13] Provinciali M, Smorlesi A (2005). Immunoprevention and immunotherapy of cancer in aging. Cancer Immunol. Immunother..

[14] Sharma S, Dominguez AL, Lustgarten J (2006). Aging affect the anti-tumour potential of dendritic cell vaccination, but it can be overcome by co-stimulation with anti-OX40 or anti-4-1BB. Exp. Gerontol..

[15] Gravekamp C (2011). The impact of aging on cancer vaccination. Curr. Opin. Immunol..

[16] Pfister G (2006). Naïve T cells in the elderly: are they still there?. Ann. N. Y. Acad. Sci..

[17] Muller WJ, Sin E, Pattengale PK, Wallace R, Leder P (1988). Single-step induction of mammary adenocarcinoma in transgenic mice bearing the activated c-neu oncogene. Cell.

[18] Kang I (2004). Age-associated change in the frequency of memory CD4+ T cells impairs long term CD4+ T cell responses to influenza vaccine. J. Immunol..

[19] Hainz U (2005). Insufficient protection for healthy elderly adults by tetanus and TBE vaccines. Vaccine.

[20] Amici A (2000). DNA vaccination with full-length or truncated Neu induced protective immunity against the development of spontaneous mammary tumours in HER-2/neu transgenic mice. Gene Therapy.

[21] Lollini PL, Cavallo F, Nanni P, Forni G (2006). Vaccines for tumour prevention. Nat. Rev. Cancer.

[22] Gravekamp C, Jahangir A (2014). Is cancer vaccination feasible at older age?. Exp. Gerontol..

[23] Utsuyama M (1992). Differential age-change in the numbers of CD4+CD45RA+ and CD4+CD29+ T cell subsets in human peripheral blood. Mech. Ageing Dev..

[24] Smorlesi A (2006). Evaluation of different plasmid DNA delivery systems for immunization against HER2/neu in a transgenic murine model of mammary carcinoma. Vaccine.

[25] Provinciali M (2009). Immunosenescence and cancer vaccines. Cancer Immunol. Immunother..

[26] Pierpaoli E (2013). Effect of annatto-tocotrienols supplementation on the development of mammary tumours in HER-2/neu transgenic mice. Carcinogenesis.

[27] Pierpaoli E (2015). Antiangiogenic and antitumour activities of berberine derivative NAX014 compound in a transgenic murine model of HER2/neu-positive mammary carcinoma. Carcinogenesis.

[28] Quaglino E (2004). Electroporated DNA Vaccine Clears Away Multifocal Mammary Carcinomas in Her-2/*neu* Transgenic Mice. Cancer Res..

[29] Curcio C (2008). DNA immunization using constant-current electroporation affords long-term protection from autochthonous mammary carcinomas in cancer-prone transgenic mice. Cancer Gene Ther.

[30] Rovero S (2000). DNA vaccination against rat her-2/Neu p185 more effectively inhibits carcinogenesis than transplantable carcinomas in transgenic BALB/c mice. J. Immunol..

[31] Stein P (2011). Regulatory T cells and IL-10 independently counterregulate cytotoxic T lymphocyte responses induced by transcutaneous immunization. PLoS One.

[32] Espinoza Mora MR (2014). Depletion of regulatory T cells augments a vaccine-induced T effector cell response against the liver-stage of malaria but fails to increase memory. PLoS One.

[33] Jagger A, Shimojima Y, Goronzy JJ, Weyand CM (2014). Regulatory T cells and the immune aging process: a mini-review. Gerontology.

[34] Bueno, V., Sant’Anna, O. A. & Lord, J. M. AGE **36**(6), 9729 doi:10.1007/s11357-014-9729-x. (2014). 10.1007/s11357-014-9729-x PMC4233024 25399072

[35] Yan J (2014). Novel and enhanced anti-melanoma DNA vaccine targeting the tyrosinase protein inhibits myeloid-derived suppressor cells and tumour growth in a syngeneic prophylactic and therapeutic murine model. Cancer gene Ther..

[36] Donnini A, Re F, Orlando F, Provinciali M (2007). Intrinsic and Microenvironmental Defects Are Involved in the Age-Related Changes of Lin_c-kit_Hematopoietic Progenitor Cells. Rejuven. Res.

