# Supplementary material for: Booster immunizations with DNA plasmids encoding HER-2/neu prevent spontaneous mammary cancer in HER-2/neu transgenic mice over life span
Source: Sci Rep. 2017 Jun 8;7:3078. doi: 10.1038/s41598-017-03286-8 (PMC5465096; doi:10.1038/s41598-017-03286-8)
Supplement: Supplementary file 1 — Supplementary Information [file 41598_2017_3286_MOESM1_ESM.pdf]

Booster immunizations with DNA plasmids encoding HER-2/neu prevent spontaneous mammary cancer in HER-2/neu transgenic mice over life span

Mauro Provinciali*, Alessandra Barucca, Fiorenza Orlando, Elisa Pierpaoli

Advanced Technology Center for Aging Research, Scientific Technological Area, IRCCS-INRCA, Ancona, Italy

Correspondence: Mauro Provinciali, MD, Advanced Technology Center for Aging Research, Scientific Technological Area, IRCCS-INRCA, Ancona, ITALY. E-mail: m.provinciali@inrca.it

Disclosure of potential conflicts of interest: The authors declare no potential conflicts of interest.

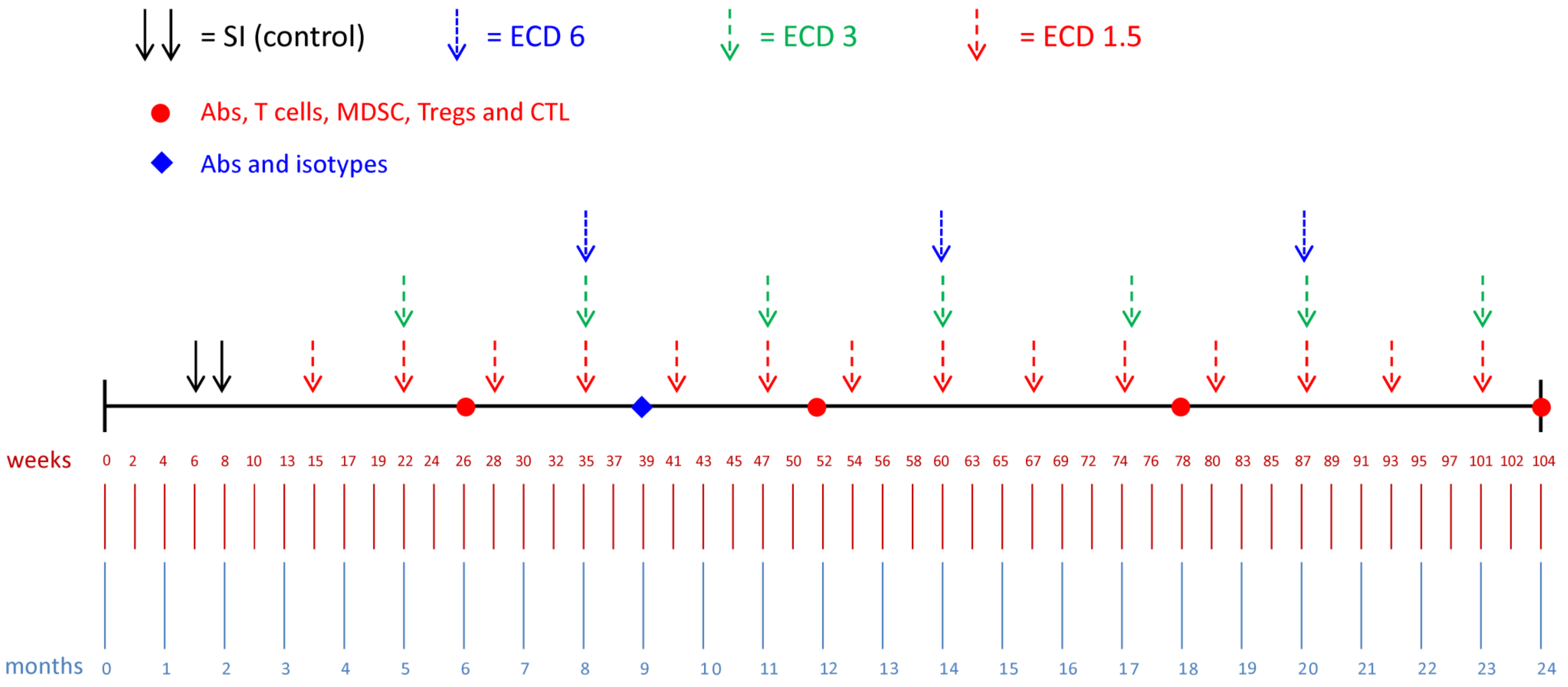
= SI (control)
= ECD 6
= ECD 3
= ECD 1.5
Abs, T cells, MDSC, Tregs and CTL
Abs and isotypes
weeks
0 2 4 6 8 10 13 15 17 19 22 24 26 28 30 32 35 37 39 41 43 45 47 50 52 54 56 58 60 63 65 67 69 72 74 76 78 80 83 85 87 89 91 93 95 97 101 102 104
months
0 1 2 3 4 5 6 7 8 9 10 11 12 13 14 15 16 17 18 19 20 21 22 23 24
